# Safety and adherence of pressure garment therapy in children with upper limb unilateral cerebral palsy. Results from a randomized clinical trial ancillary analysis

**DOI:** 10.3389/fped.2023.1043350

**Published:** 2023-03-21

**Authors:** Laurent Béghin, Yasser Mohammad, Séverine Fritot, Guy Letellier, Sixtine Masson, Yann Zagamé, Catherine Donskoff, Mathide Toussaint-Thorin, Laurence Gottrand

**Affiliations:** ^1^ CIC 1403 – Clinical Investigation Center. Lille University Hospital Inserm, Lille, France; ^2^ University of Lille, Inserm, CHU Lille, U1286 - INFINITE - Institute for Translational Research in Inflammation, Lille, France; ^3^ Pediatric Physical Medicine and Rehabilitation Center APF, Creil, France; ^4^ Rehabilitation Center, Beaumont sur Oise, France; ^5^ Physical Medicine and Rehabilitation Department, CHU Amiens, Amiens, France; ^6^ Pediatric Physical Medicine and Rehabilitation Center (ESEAN-APF), Nantes, France; ^7^ Physical Medicine and Rehabilitation Center APF (Centre Marc Sautelet), Villeneuve-d’Ascq, France; ^8^ Medical Z®, F-37208, Saint Avertin, France; ^9^ Physical Medicine and Rehabilitation Department, Paul Dottin Center, Ramonville-Saint-Agne, France; ^10^ Pediatric Physical Medicine and Rehabilitation Center, CHU Reims, Reims, France

**Keywords:** unilateral upper limb cerebral palsy, pressure garment therapy, safety, adherence, children

## Abstract

**Background:**

This study was conducted to assess the safety and adherence of the use of a PGT (Pressure Garment Therapy) Lycra® sleeve to treat upper limb unilateral cerebral palsy (UCP) in children.

**Methods:**

This study was conducted as a prospective, placebo-controlled, double-blinded, randomized monocenter study. Included in the study were 58 UCP children, 49 of whom were analyzed. 25 children (mean age 6.6 ± 1.6 years; 12 girls) were allocated to the active group vs. 24 (mean age 6.7 ± 1.6 years; 10 girls) in the placebo group. The intervention consisted of an active PGT Lycra® arm sleeve manufactured to generate a homogeneous pressure ranging from 15 to 25 mmHg. The placebo PGT Lycra® sleeve was manufactured to generate a homogeneous pressure under 7 mmHg. The time of wearing period was set at 3 h/day at minimum and 6 h/day at maximum, over the course of 6 months. The main outcome measures were safety outcomes including the number and intensity of Adverse Events of Special Interest (AESIs). AESIs were defined as adverse events imputable to compressive therapy and Lycra® wearing. Level of adherence was expressed in percentage of number of days when the sleeve was worn for at least 3 h per day compared to length of duration in days (start and end date of wearing period).

**Results:**

Frequency of AESIs were very low and no different between groups (4.12 ± 11.32% vs. 1.83 ± 3.38%; *p* = 0.504). There were no differences in adherence (91.86 ± 13.86% vs. 94.30 ± 9.95%; *p* = 0.425).

**Conclusion:**

The use of PGT Lycra® arm sleeve in children with UCP is safe and well-tolerated with a very good adherence. The low rate of AESIs is promising for further randomized clinical trials on efficacy.

## Introduction

Cerebral Palsy (CP) is a common neurodevelopmental disorder in childhood that affects 2 per 1,000 live births (1, 2). CP is caused by non-progressive lesions in the immature brain occurring before, during, or after birth. The main symptoms of unilateral CP (UCP) are spasticity and limited upper arm/hand function, affecting the child's ability to use their hands (3, 4). Sensory motor impairment compromises the development of manual dexterity, which is crucial for performing usual daily activities. Recent reviews and meta-analyses have provided evidence-based arguments to guide management of UCP such as constraint-induced therapy, occupational therapy, botulinum neurotoxin injection, hand-splints, and task-specific training (5–8). The use of Pressure Garment Therapy (PGT) has also been used to treat upper arm UCP or other similar conditions in adults (9, 10). PGT was performed using synthetic polyurethane/elastane fiber used in the confection of tailor-made close-fitting garments as a medical device. The most common synthetic polyurethane/elastane fiber used is Lycra®, which is commercially available as a dynamic sleeve that uses tensile properties of polyurethane to generate torsion, correct muscle force imbalance across joints, optimize muscle length and functional positioning, and provide a constant/homogenate compression with neutral heat on the concerned UCP arm (11, 12). These properties are used to improve postural alignment, joint stability, and movement efficiency and enhance posture, balance, coordination, gross motor function, hand function, and gait of upper arm in children with upper arm UCP or other health conditions (8, 9, 13–18).

The role of Pressure Garment Therapy (PGT) using a Lycra® arm sleeve is to increase sensory and proprioceptive awareness and reduce abnormal tone of the concerned body part (19). These properties may improve hand functional performance of children with UCP (20). PGT could be performed by an arm dynamic sleeve manufactured using Lycra®. Lycra® sleeves have been widely used for burn therapy (21, 22) but their efficacy as a PGT is unknown in UCP (20). The mechanical properties of Lycra® sleeves and their usability have been established in studies involving healthy and hemiplegic adult subjects (11, 23). To date, there is no data of safety and adherence of PGT Lycra® arm sleeves in UCP children (24). The knowledge of PGT safety and adherence is crucial to successfully understand the efficacy of this device. Indeed, if safety and adherence are low, the development of this device should be discontinued without a randomized clinical trial.

The aim of this study was to assess the safety and adherence of the use of a PGT Lycra® sleeve to treat upper limb UCP in children.

## Methods

### Study settings and sample

This ancillary analysis is a part of the previously described (20) multicenter (seven study sites), prospective, double-blinded, randomized, placebo-controlled clinical trial. The present study is an ancillary analysis of 58 children included in one institutional specialized rehabilitation unit.

**Figure 1:**
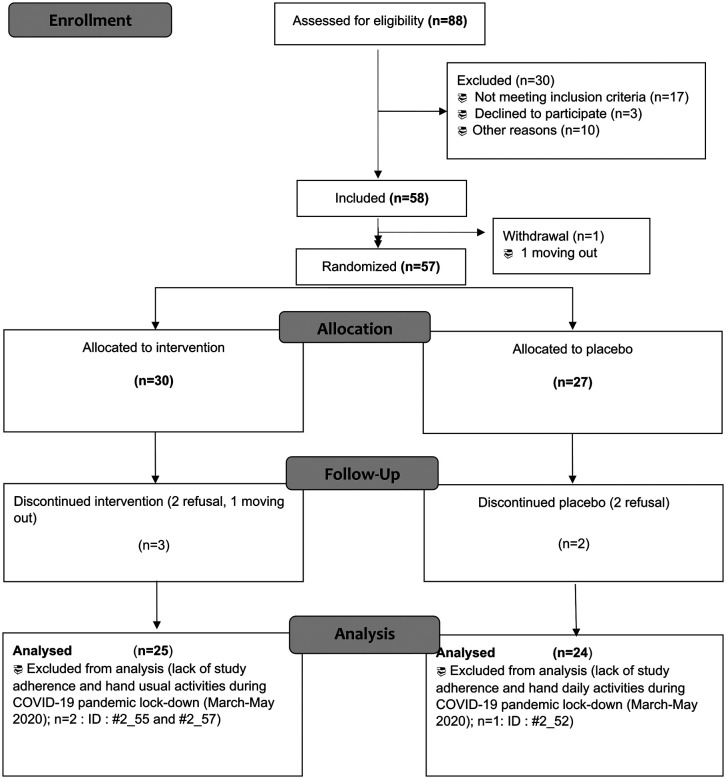
CONSORT flow diagram.

Each child with CP was included after a physical examination to ensure the inclusion criteria were met. The criteria included children with ante-natal or peri-natal upper arm UCP, aged 5–10 years old, with social insurance, and with parental written informed consent. The exclusion criteria were allergy to Lycra®, contraindication to pressure therapy (e.g., skin lesions or allergic contact dermatitis), behavior or speech troubles, treatment with botulinum neurotoxin on the involved arm within the preceding 4 months, or predictable lack of compliance. The consort flow diagram of the recruitment process and allocation in the study site is described in Figure 1. Enrollment into the study was started in January 2013 and finished in October 2020. Of the 58 included UCP children, 49 were analyzed for the purpose of this ancillary study. Clinical characteristics and classification of UCP children were carried out according to Mandaleson et al. (25).

### Study intervention procedure

Each PGT Lycra® sleeve was measured by a well-trained physiotherapist (trained by a specialist from the manufacturer) according to manufacturer's instructions. Size segments were measured at standard points of reference for circumferential measurements (Supplementary Figure S1). It was a tailor-made sleeve which covered the arm from the axilla to half of the palm and the thumb, without covering other fingers (20). Lycra® sleeves were manufactured for each UCP child according to a randomization list within 1 week. A randomization sequence 1:1 ratio was performed by an independent statistician using computer-generated random numbers structured in blocks generated by PROC Plan SAS software version 9.4 (SAS Institute Inc., Cary, NC). All participants and research staff were blinded regarding the randomization allocation during the whole duration of the trial, as were the parents, physiotherapists, and physicians involved in the child's care. The active sleeve and the placebo sleeve were the exact same appearance. All sleeves were made of Coolmax® textil UPF 40+ (75% of Polyester Coolmax and 25% of Elasthame Lycra®) and skin color using a using 6-way stretch fabrication. The active PGT Lycra® sleeve was manufactured to generate a homogeneous pressure ranging from 15 to 25 mmHg. The pressure was ranked as a class I/II quality assurance French standard medical compression arm sleeve (26, 27). The placebo PGT Lycra® sleeve was manufactured to generate a homogeneous pressure with minimized reduction targeting a 5/7 mmHg. The time of wearing period was set at 3 h/day at minimum and 6 h/day at maximum, over the course of 6 months.

In order to check the compressive properties of the Lycra® sleeve, a subgroup of sleeves was manufactured in duplicate (i.e., the first one for the UCP children included in the study, the second for laboratory compression testing). This subgroup consisted of the 10 first UCP children sleeve measurements included in this study. The compressive properties of these sleeves were measured in a laboratory using an extensometer with normalized compression force scale (Extensometer NF G 30 102-B, EMI Development, Paris, France).

In both groups, the child was asked to wear the PGT Lycra® sleeve (active or placebo) for at least 3 h per day, every day, for 6 months (20). The PGT Lycra® sleeve was to be worn during usual daily activities, especially in activities involving bimanual performances, and during rehabilitation sessions. Each patient's therapists (physiotherapists and occupational therapists) were informed of the child's enrollment in a trial and asked to follow instructions of harmonization for rehabilitation, but there was no major modification of the usual rehabilitation care. Written general recommendations were provided to guide rehabilitation:

- Stimulation of proprioceptive function: analytic proprioception stimulation, ground bearing and weight-bearing transfers, installation quality and symmetry, mirror feedback, and dynamic proprioception stimulation (opposition and pushing games, moving of heavy objects, etc.).
- Stimulation of active mobility, on proximal and distal levels of the hemiplegic side, with static shoulder and arm; hand aiming; and approach, grip, and release exercises.
- Stimulation of bimanual coordination during daily activities, developing assisting hand capacities and passing from one hand to the other.

Additional personalized recommendations were added regarding the child’s state of development and actual capacities of the upper extremity.

### Study variables

The primary objective of this study was to evaluate the safety of a PGT Lycra® sleeve worn over 6 months on the upper arm of children with UCP. Safety outcomes included the number and intensity of Adverse Events of Special Interest (AESIs). AESIs were defined as adverse events imputable to compressive therapy and Lycra® wearing, localized at the arm. AEIs were classified into two subgroups: (i) cutaneous events linked to Lycra® wearing and (ii) compression events linked to compressive therapy. According to the device classification panel from the FDA, the PROPENSIX PGT Lycra® sleeve is a class I medical device. In this context and according to French regulations, it was not mandatory to address a Medical Device Reporting process and a Data Safety Monitoring Board report to conduct this study.

Parents were asked to report AESIs and other problems occurring during the Lycra® sleeve pressure garment wearing period daily in a parental self-report diary logbook (Supplementary Figure S2 Log diary) as a measurement tool. This type of self-report questionnaire has already been used in previous studies for AESI monitoring (28). Diary logbooks were carefully checked by an investigator before physical examination at each hospital visit (i.e., 3 and 6 months) using a structured interview as described Knowles et al. (29). AESIs were classified *a posteriori* by an investigator using Bégaud et al. classification (minor/moderate/serious) usually used for drug clinical trial reports (30). Cutaneous events included itchy contact dermatitis, red skin rash, and spots; compression events included mechanical swelling, pain in the arm, “Blue hand”, tingling, discomfort, sore thumb, and compliant from child of tightness. Frequency of AESIs were computed as a percentage of occurred days of AESIs from the total wearing time.

Systolic blood pressure (SBP) and diastolic blood pressure (DBP) were measured as a safety outcome using an oscillometric device KYPIA COLSON (COLSON®, Paris, France) with a pediatric bladder following the recommendations of the British Hypertension Society (31) and recommendations for children populations (32). SBP and DBP were measured on the hemiplegic arm of UCP children without the Lycra® sleeve pressure garment. SBP and DBP were measured in a sitting position after a 10 min rest in a quiet room after sleeve removal at least 10 min before measurement. The duration of 10 min of sleeve removal before BP measurement was considered enough time to not affect BP data (33).

Level of adherence was assessed using a conventional paper-based method (34–36). The diary logbook collected daily amount of Lycra® sleeve pressure garment wearing period in number of hours and the reason of non-adherence if the sleeve was worn for under 3 h per day. Level of adherence was expressed in percentage of number of days when the sleeve was worn for at least 3 h per day compared to length of duration in days (start and end date of wearing period). Frequency of reasons of non-adherence were computed as percentage of occurred days of AESIs from the total wearing time.

Anthropometrics and neurologic examination acquisition data were previously described by Gerard et al. (20). Weight was measured in underwear, with shoes removed, using an electronic scale (SECA® 861, SECA®, Birmingham, UK) to the nearest 0.1 kg. Height was measured with shoes removed using a telescopic height measuring instrument (SECA® 225) to the nearest 0.1 cm. Functional profile of the child was classified according to Gross Motor Function Classification Scale (GMFCS): type I–V (37) and Manual Abilities Classification System (MACS) type I–V (38). CP etiology was classified according to previous studies (39, 40) according to *in utero* or perinatal events (41).

Physical examination (standard clinical exam and neurologic exam) was performed by the same investigator throughout the study period.

Data were entered in electronic Case Report Forms (eCRF). The eCRF was developed using Ennov Clinical© software. Data were checked by the data management team of the data monitoring department of the trial sponsor using the predefined rules of quality assurance.

### Statistical analysis

Data are presented as frequency (percentage) for categorical variables and mean ± standard deviation (SD) for continuous variables. Normality of distribution was checked graphically with the Shapiro–Wilk test. Comparisons between groups (intervention vs. placebo) were compared using Khi2 test for categorical variables and Student's *t* test for continuous variables. ANOVA for repeated measures was used for longitudinal data analysis. All statistical tests were done at the two-tailed *α* level of 0.05. Data were analyzed with SAS software version 9.4 (SAS Institute Inc., Cary, NC).

## Results

Using the data from the first 10 children with CP Lycra® sleeve pressure garment measurements, a second set of 10 sleeves were manufactured for compression laboratory testing. Data of compressive properties of these sleeves measured in the laboratory were obtained for four active PGT Lycra® sleeves and six placebo PGT Lycra® sleeves. The mean and standard deviation of compression was 17 ± 1 mmHg for active vs. 7 ± 1 mmHg for placebo.

**Figure 2:**
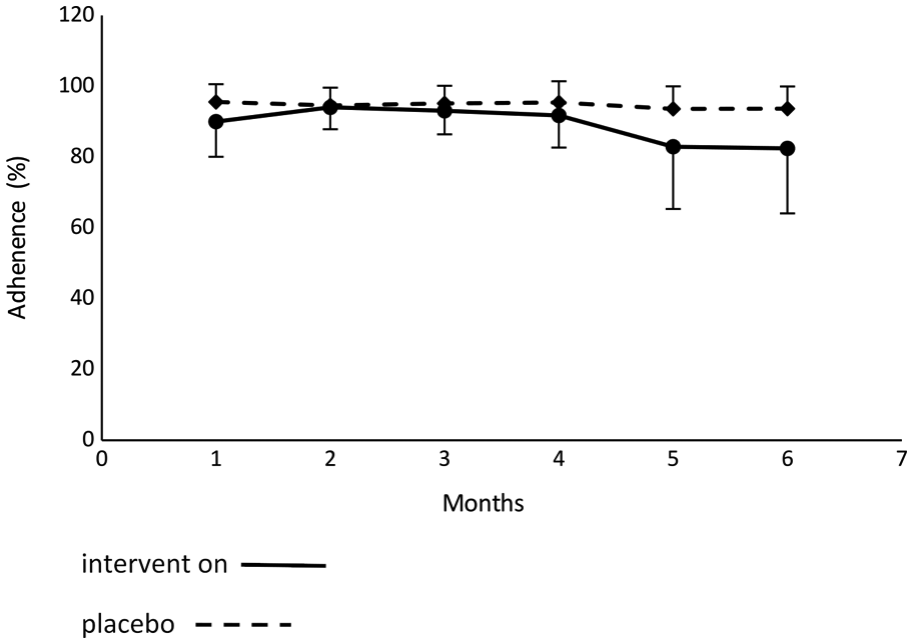
Evaluation of PGT Lycra® arm sleeve adherence throughout the study period.

Figure 1 presents the CONSORT flow diagram of this ancillary study. Of the 58 included UCP children, 49 were analyzed (25 intervention vs. 24 placebo), which allowed a well-balanced and sufficiently powerful statistical analysis between groups. Clinical characteristics of UCP children at baseline are presented in Table 1. There was no difference in anthropometrics, etiology, and neurological contexts between groups. Neither wearing period/daily time duration nor adherence of over 90% were different between groups. Mean adherence is shown in Table 2 and slightly lower in the intervention group (91.86 ± 13.58%) than in the placebo group (94.3 ± 6.95%), mainly due to four subjects with very low adherence under the expected 80% (64.49% for #2_018; 66.25% for #2_036; 54.0% for #2_44 and 66.13% for #2_051). Figure 2 presents the evolution of adherence throughout the study period. There was no difference of evolution pattern between groups and no decrease of observance throughout the study period. Table 3 presents frequency reasons of non-adherence of sleeve. The most common reason of non-adherence was AESIs provoked by Lycra® sleeve wearing; there was no difference between groups. Other reasons for non-adherence were all under 1.3% and were not different between groups. During the study period, it was necessary to do a replacement of the Lycra® sleeve (needed a new measurement and manufacturing) with a new one in three intervention subjects (#2_006; #2_009; #2_024) and two placebo subjects (#2_010; #2_024).

**Table 1:** Clinical characteristics of UCP children at baseline between allocation groups.

	Intervention group (*n* = 25)	Placebo group (*n* = 24)	*p*
Anthropometrics			
Gender M/F	13/12	14/10	0.904
Age (years)	6.6 ± 1.6	6.7 ± 1.6	0.876
Weight (kg)	23.8 ± 8.6	19.5 ± 3.5	0.496
Height (cm)	122.2 ± 10.8	124.5 ± 11.2	0.533
Neurologic context			
Etiology (FS/PS/PL/CM/II/PT/UK)	6/8/1/2/2/1/1	10/5/2/4/2/0/1	0.743
UCP type (S/A/D/M)	17/0/3/4	17/1/3/3	0.894
UCP laterality (left/right)	9/16	10/12	0.936
GMFCS level (I, II, III, IV, V)	10/14/1/0/0	9/14/0/1/0	0.567
MACS (I, II, III, IV, V)	2/9/11/3/0	4/6/12/2/0	0.675

Values are in mean ± standard deviation; Etiology codes: FS/PS/PL/CM/II/PT/UK (Fetal Stroke/Perinatal Stroke/Periventricular Leukomacia/Congenital Malformation/In utero Infection/Perinatal Traumatic head injury/UnKnown); UCP, unilateral cerebral palsy; UCP type: S/A/D/M: Spastic/Ataxic/Dystonic/Mix; GMFC, gross motor function classification system; MACS, manual ability classification system.

**Table 2:** Lycra® sleeve wearing period/daily time duration, mean frequency of adherence and sleeve replacement numbers between UCP children allocation groups.

	Intervention group (*n* = 25)	Placebogroup (*n* = 24)	*p*
Wearing duration period (days)	199.32 ± 73.46	207.17 ± 32.35	0.857
Daily duration (hours)	4.24 ± 0.89	4.51 ± 1.03	0.345
Adherence (%)	91.86 ± 13.86	94.30 ± 9.95	0.425
Sleeve replacement (*n*)	3*	2**	0.825

Values are in mean ± standard deviation.

* For 3 subjects #6, #9 and #18.

** For 2 subjects #10 and #24.

**Table 3:** Frequency reasons of non-adherence of Lycra® sleeve wearing between UCP children allocation groups.

	Intervention group (*n* = 25)	Placebo group (*n* = 24)	*p*
	%	%	
AEIs	1.31 ± 4.53	0.14 ± 0.70	0.218
Oversight for holidays	1.29 ± 4.53	0.84 ± 2.61	0.679
Child refusal	0.95 ± 3.96	0.06 ± 0.16	0.276
Washing sleeve	0.74 ± 3.62	0.09 ± 0.32	0.383
Oversight at school	0.79 ± 1.81	1.22 ± 3.47	0.588
Mechanical problem	0.73 ± 2.59	0.06 ± 0.29	0.213
Sport activity	0.03 ± 0.13	0.27 ± 0.85	0.165
“Let go”	0.03 ± 0.13	0.08 ± 0.28	0.438
Others	2.10 ± 3.80	2.14 ± 4.66	0.976
Total	7.96 ± 1.87	4.90 ± 1.64	0.888

Values are means ± SD; AEI, adverse events of interest; Others: moving from an another home for separated parents; canicular temperatures, unknown.

**Figure 3:**
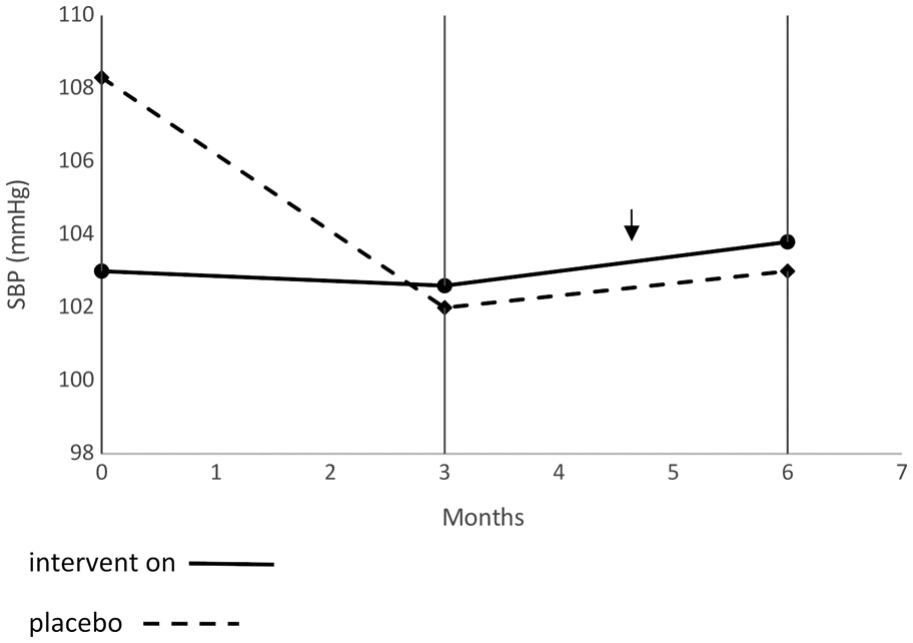
Evaluation of systolic blood pressure throughout the study period.

**Figure 4:**
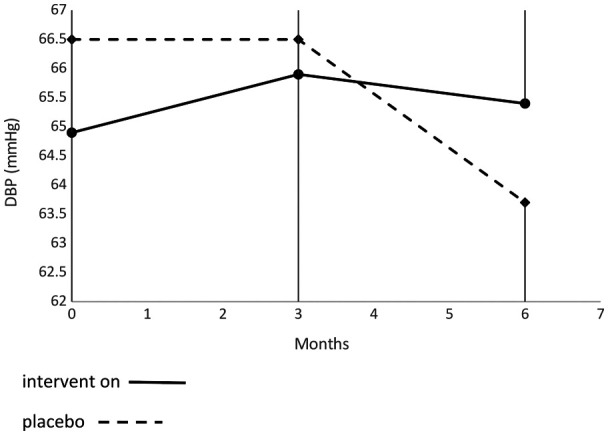
Evaluation of diastolic blood pressure throughout the study period.

Table 4 presents the frequency of AESIs classified as minor between groups. Mean AESI was 4.11% in the active group vs. 0.5% in the placebo group. The most common AESI observed was mechanical swelling in the intervention group (2.15% vs. 0% in placebo group). Others AESIs were under 1% and were not different between groups. Figures 3, 4 present evolution of SBP and DBP between groups, respectively, without any difference between groups.

**Table 4:** Frequency of adverse events of interest between UCP children allocation groups.

	Intervention group (*n* = 25)*	Placebo group (*n* = 24)**	*p*
	%	%	
Mechanical swelling	2.15 ± 6.80	0.00 ± 0.00	0.528
Pain at arm	0.41 ± 0.64	0.49 ± 0.99	0.481
“Blue hand”	0.12 ± 0.39	0.12 ± 0.24	0.939
Tinglings	0.06 ± 0.19	0.00 ± 0.00	0.029
Unconfortable	0.00 ± 0.00	0.12 ± 0.24	0.139
Sore thumb	0.00 ± 0.00	0.12 ± 0.25	0.139
Complain	0.00 ± 0.00	0.12 ± 0.24	0.139
Tight	0.00 ± 0.00	0.24 ± 0.48	0.529
Itchy contact dermatitis	0.78 ± 2.21	0.37 ± 0.47	0.260
Red skin rash	0.52 ± 0.85	0.00 ± 0.00	0.139
Spots	0.05 ± 0.16	0.25 ± 0.49	0.207
**Total**	**4.12 ± 11.32**	**1.83 ± 3.38**	0.504

Values are in mean ± standard deviation.

* For 8 subjects #18, #23, #30, #35, #40, #44, #51 and #54.

** For 4 subjects #31, #42, #49 and #54.

## Discussion

Mechanical properties of Lycra® sleeves have been established in studies involving healthy and hemiplegic adult subjects (11, 23) and were shown to be well-tolerated without any serious adverse events. This study is the first assessing PGT in children with upper limb UCP in free living conditions (during school, playing, and eating activities, etc.).

All children in this study were of a normal weight with a stable weight throughout the study period (data not shown), which allowed a good analysis of study adherence. Neurologic context and etiology of UCP was varied and were well-balanced between groups, which allowed a good reproducibility of using PGT Lycra® sleeve for future randomized intervention studies.

Compression of the placebo Lycra® sleeve manufactured in duplicate reached the targeted planned placebo compression of 7 mmHg. This minimal compression was sufficient to be as close as possible to the double-blind methodology and as the best sleeve fit as possible. Compression of the active Lycra® sleeve was the same (17 mmHg) that Barss et al. (42) used to enhance proprioception.

The level of adherence was very good and was more than 80% for most patients throughout the study period. The very good level of adherence observed was due to: (i) the Medical Z PGT Lycra® sleeve not covering a large part of the body and allowing the fingers to be free, the “skin” color being acceptable for children, and the Coolmax® textil was comfortable; (ii) the high accuracy of Lycra® sleeve measurement by well-trained physiotherapists and a well-designed template (Supplementary Figure S2) allowed the garments to fit the arm anatomy well and meant the sleeve was easy to put on; (iii) a low rate of AESI frequency; and (iv) the child was able to carry out their usual activities, rhythmed by the usual environment, in a context of confidence. Non-adherence ratio observed in this study was very low and mostly focused on four subjects from intervention group. This low level of adherence observed in the intervention group was due to AESIs such as mechanical swelling and itchy contact dermatitis (subjects #2_ 018, #2_036, #2_044 and #2_051 allocated in intervention group). AESIs were not a cause of non-adherence since their frequency reached 4.11%, while AEIs frequency in cause of non-observance was only 1.31%. AESIs quickly occurred during the early period of the study (data not shown).

SBP and DBP were within normal ranges according to recommendations for child populations (32) throughout the study period. This observation confirms the safety and tolerance of using Medical Z PGT Lycra® sleeves for a long period (i.e., 6 months) in children with UCP. The wearing period was set as 6 months and was enough time to detect most AESIs. AESI frequency was very low and the most common was mechanical swelling, especially in the active group. Pain from sleeve wearing frequency was very low and not in accordance with the concept of “No pain, no gain” previously described (43). The AESI Itchy contact dermatitis was also very low, provoking some minor skin irritations.

This study has several strengths: (i) 6 months was a sufficient therapy duration, (ii) the high level of threshold dose (i.e., 3–6 h per day) was similar to previous studies (6), abd (iii) the homogenous compression allowed detection of most AESIs. This study also has several limitations: (i) AESIs and adherence data were initially collected by parents who could have under/overestimated AESIs as previously described (44, 45), (ii) AESIs were difficult to be classified by investigators since no clear AE classification system exists for medical devices (46, 47), and (iii) data on usability and satisfaction was not provided as in previous published studies (48). Despite this, the use of Medical Z PGT Lycra® sleeves is promising since six parents spontaneously asked for compassionate use of this device at the end of the study. This strength is very useful as children with UCP are often limited in performing daily living activities (49). In conclusion, this ancillary PROPENSIX study will provide arguments about the feasibility of PGT Lycra® arm sleeves in children with UCP. The adherence was very good, with a low rate of AESIs. Adherence data will be useful to analyze the primary aim of the PROPENSIX study using intention to treat process (50). The PGT Lycra® arm sleeve is quickly manufactured and can be delivered within 1 week. A large sample size (*n* > 100) multicenter randomized clinical trial (RCT) is currently ongoing to assess the efficacy of this device on hand performance (20). The use of adherence ratio is useful for analyzing RCT in per protocol and intention to treat statistical analysis. The low rate and accurate description of AESIs is promising for further RCT and its usability in the real world. Moreover, this study has strengthened the transparency of information for new users as requested by the new EU Medical Device Regulations, which was fully enforced in 2022.

## Data Availability

The raw data supporting the conclusions of this article will be made available by the authors, without undue reservation.
