# Supplementary figures and images for: Safety and adherence of pressure garment therapy in children with upper limb unilateral cerebral palsy. Results from a randomized clinical trial ancillary analysis
Source: Front Pediatr. 2023 Mar 21;11:1043350. doi: 10.3389/fped.2023.1043350 (PMC10071041; doi:10.3389/fped.2023.1043350)

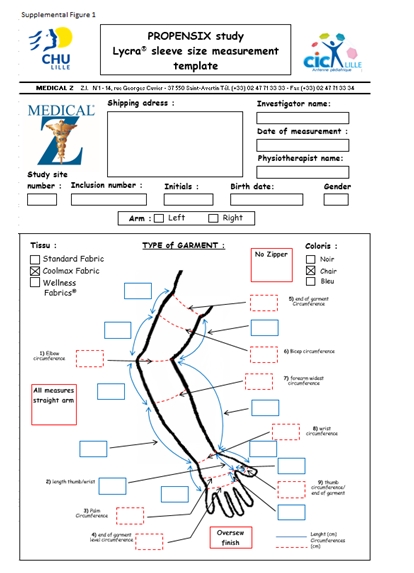

Supplement: Supplementary file 1 [file Image1.jpeg]

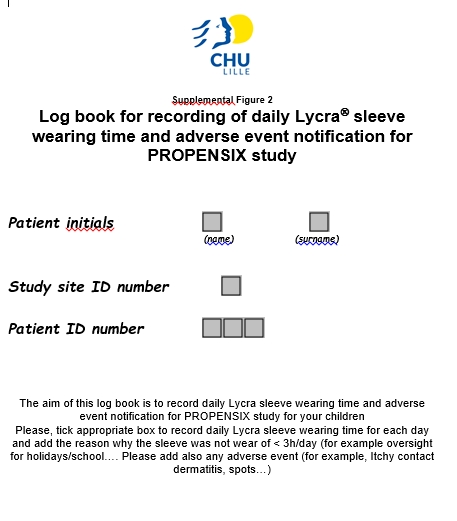

Supplement: Supplementary file 2 [file Image2.jpeg]

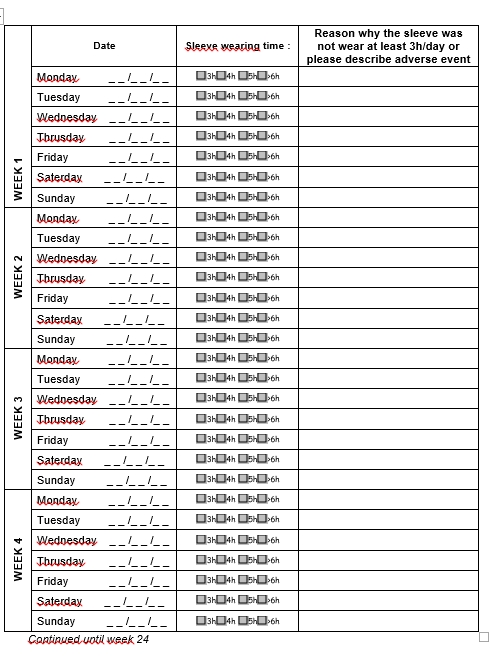

Supplement: Supplementary file 3 [file Image3.jpeg]
